# Cytoplasmic regulation of chloroplast ROS accumulation during effector-triggered immunity

**DOI:** 10.3389/fpls.2023.1127833

**Published:** 2023-01-30

**Authors:** Jianbin Su, Walter Gassmann

**Affiliations:** Division of Plant Science and Technology, Christopher S. Bond Life Sciences Center, and Interdisciplinary Plant Group, University of Missouri, Columbia, MO, United States

**Keywords:** reactive oxygen species (ROS), effector-triggered immunity (ETI), mRNA decay, cotranslational mRNA decay, Rubisco-containing body (RCB)

## Abstract

Accumulating evidence suggests that chloroplasts are an important battleground during various microbe-host interactions. Plants have evolved layered strategies to reprogram chloroplasts to promote *de novo* biosynthesis of defense-related phytohormones and the accumulation of reactive oxygen species (ROS). In this minireview, we will discuss how the host controls chloroplast ROS accumulation during effector-triggered immunity (ETI) at the level of selective mRNA decay, translational regulation, and autophagy-dependent formation of Rubisco-containing bodies (RCBs). We hypothesize that regulation at the level of cytoplasmic mRNA decay impairs the repair cycle of photosystem II (PSII) and thus facilitates ROS generation at PSII. Meanwhile, removing Rubisco from chloroplasts potentially reduces both O_2_ and NADPH consumption. As a consequence, an over-reduced stroma would further exacerbate PSII excitation pressure and enhance ROS production at photosystem I.

## Introduction

Chloroplasts are the energy center where solar energy is converted into chemical energy *via* the photosynthetic electron transport chain (PETC). Oxygenic photosynthesis unavoidably generates a large amount of reactive oxygen species (ROS) in the form of singlet oxygen (^1^O_2_), superoxide (O_2_^•-^), hydroxyl radicals (OH^•^) and hydrogen peroxide (H_2_O_2_) (Foyer and Hanke, 2022; Li and Kim, 2022). Because of the coordination between photosynthetic control and ROS scavenging systems, chloroplastic ROS (cROS) are maintained at a relatively low level (Foyer and Hanke, 2022; Li and Kim, 2022). cROS accumulation has been observed in pattern-triggered immunity (PTI), effector-triggered immunity (ETI) and various types of host-microbe interactions (Liu et al., 2007; Dong and Chen, 2013; de Torres Zabala et al., 2015; Lu and Yao, 2018; Shang-Guan et al., 2018; Su et al., 2018; Kuźniak and Kopczewski, 2020; Kachroo et al., 2021; Littlejohn et al., 2021). Based on the observation that ETI triggers cROS accumulation whereas effectors of a virulent pathogen suppress this response (de Torres Zabala et al., 2015; Su et al., 2018), it has been widely accepted that cROS is essential for robust ETI (Lu and Yao, 2018; Sowden et al., 2018; Kuźniak and Kopczewski, 2020; Kachroo et al., 2021; Littlejohn et al., 2021). It is worth noting that cROS promotes susceptibility to necrotrophic pathogens (Rossi et al., 2017).

In most cases known to date, ETI is initiated by the activation of intracellular nucleotide-binding leucine-rich repeat receptors (NLRs). According to their biological functions, NLRs are classified into sensor NLRs and helper NLRs (Adachi et al., 2019; Feehan et al., 2020). Upon activation, the majority of sensor NLRs further activate helper NLRs to execute ETI. Activated helper NLRs oligomerize at the plasma membrane to form Ca^2+^ channels, leading to cytoplasmic Ca^2+^ influx (Jacob et al., 2021.; Contreras et al., 2022). In the case of the ancient sensor NLR ZAR1, which is functionally conserved across plant species, it is the sensor NLR itself that oligomerizes to form Ca^2+^ channels (Bi et al., 2021). Cytoplasmic Ca^2+^ influx triggers multiple downstream immune responses, including activation of calcium-dependent protein kinases (CPKs) and mitogen-activated protein kinases (MPKs), ROS accumulation both at the plasma membrane and in chloroplasts, and reprogramming of the cell at transcriptional, translational, and metabolic levels (Ngou et al., 2022). Rapid chloroplast Ca^2+^ transients were observed during PTI. Activation of pattern recognition receptors in PTI also leads to cytoplasmic Ca^2+^ influx, in this case mediated by plasma membrane-resident Ca^2+^ channels of the cyclic nucleotide-gated channel (CNGC), the glutamate receptor-like (GLR) channel, and the reduced hyperosmolality induced Ca^2+^ increase (OSCA) channel families (Kim et al., 2022). This cytoplasmic Ca^2+^ influx in turn is sensed by the chloroplast-localized calcium-sensing receptor (CAS) (Nomura et al., 2012). Because *cas* mutants showed an impaired ETI-induced hypersensitive response (HR), it is highly possible that cytoplasmic Ca^2+^ influx could also alter chloroplast Ca^2+^ dynamics during ETI, which might affect cROS accumulation. This review will discuss how cROS accumulation might be regulated long-term by cytoplasmic processes after the initial events of ETI activation. Because we focus our discussion on events in the cytoplasm, other important contributions to cROS accumulation, such as the chloroplast internal PSII repair cycle and chloroplastic Ca^2+^ dynamics, are not included in this minireview.

## Photosynthetic mRNA decay during ETI

The stability of mRNA varies widely, with deduced half-lives ranging from minutes to more than 24 h (Narsai et al., 2007). The deduced half-lives for many photosynthesis-related transcripts are long-lived, such as *LHCB4.2* (t_1/2_ ≈ 4.8 h), *PSAD-2* (t_1/2_ ≈ 5.5 h), *PSBQ-2* (t_1/2_ ≈ 8.1 h), *PSBP-1* (t_1/2_ ≈ 13.4 h), *PSAH-1* (t_1/2_ ≈ 17.6 h), and *PSAK* (t_1/2_ ≈ 20.3 h). An analysis of early microarray data found that mRNA levels of photosynthesis-related genes involved in light reaction, carbon assimilation, and chlorophyll synthesis globally decrease irrespective of the type of host-microbe interaction, suggesting that global downregulation of photosynthesis-related genes is a component of defense responses (Bilgin et al., 2010). Later, drastic downregulation of photosynthesis-related transcripts was observed by activation of MPK3 and MPK6 (Su et al., 2018), two MAP kinases displaying sustained activation during ETI (Tsuda et al., 2013; Su et al., 2018). Surprisingly, many transcripts encoding subunits of PSII and PSI as well as PSII activity regulators drastically decreased after long-term MPK3/MPK6 activation or during ETI (Su et al., 2018; Yoo et al., 2020). Interestingly, genes at each step of the PSII repair cycle were found to be drastically decreased by MPK3/MPK6 activation (Su et al., 2018), for example STN8 kinase required for PSII core protein phosphorylation, metalloproteases FtsH1/2/5 and Deg family of proteases Deg5/8 involved in the degradation of damaged PSII core protein D1, and factors such as LQY1, PAM68, PSB28, LBA1, LBA2, and ALB3 involved in *de novo* synthesis and assembly of D1 into the PSII core. Thus, the authors proposed that PSII is actively damaged during ETI, which promotes the accumulation of cROS (Su et al., 2018). The drastic decrease of these otherwise highly stable transcripts suggests that photosynthesis-related transcripts are actively degraded during ETI *via* mRNA decay mechanisms.

**Figure 1:**
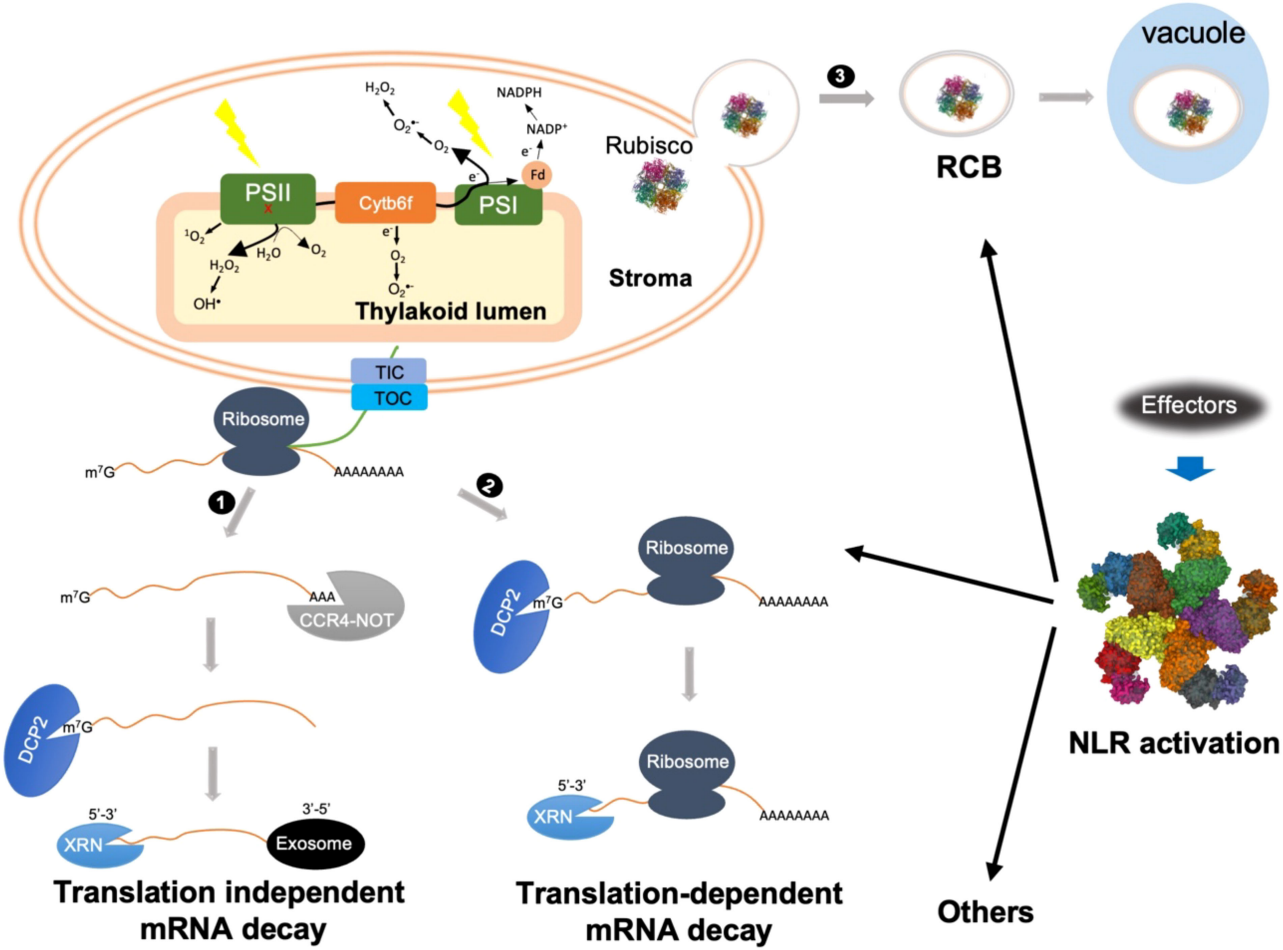
Schematic representations of possible cytoplasmic regulatory mechanisms of cROS accumulation during ETI. ETI activation might trigger both translation-dependent and -independent mRNA decay to actively degrade photosynthetic transcripts. 1) Translation-independent mRNA decay is initiated with CCR4-NOT complex-mediated deadenylation. After 5’ decapping, further degradation is carried out by either 5’-3’ or 3’-5’ decay mediated by XRN or exosome, respectively. 2) Translation-dependent mRNA decay can only occur through XRN-mediated 5’-3’ decay. Bulk mRNA decay of photosynthetic transcripts impairs the repair or turnover cycle of photosynthetic complexes, mainly PSII, resulting in ^1^O_2_ and OH^•^ production from damaged PSII, indicated by a red x. 3) NLR activation triggers autophagy-dependent formation of Rubisco-containing body (RCB) through which Rubisco as well as other stroma proteins are delivered into the vacuole for degradation. Removal of Rubisco may cause stroma overreduction and/or reduce O_2_ consumption, which facilitates electron leakage from PSI as well as cytochrome b_6_f complex (Cyt b_6_f) to O_2_, resulting in O_2_^•-^ formation at both stroma side and inside thylakoid lumen. Other cytoplasmic mechanisms might also contribute to cROS accumulation during ETI.

mRNA decay plays an important role in fine-tuning mRNA abundance. Eukaryotic mRNAs are characterized by a 5’ m^7^G cap and a 3’ poly(A) tail. The classical view on mRNA decay was thought to consist of a step-wise process involving mRNA disassociation from ribosomes, progressive removal of the poly(A) tail, 5’ decapping, and exonucleolytic digestion in either the 3’-to-5’ or 5’-to-3’ direction (Abbasi et al., 2013; Łabno et al., 2016; Zhang and Guo, 2017; Li et al., 2018) (**Figure 1**). Specifically, mRNA catabolism is typically initiated with 3’ poly(A) deadenylation by the 3’-5’ poly(A)-specific ribonuclease (PRAN) complex and the carbon catabolite repression 4-negative on TATA-less (CCR4-NOT) complex. Then, deadenylated mRNA undergoes either 3’-to-5’ decay by the RNA exosome, a multi-subunit exonuclease complex, or 5’-to-3’ decay mediated by the XRN family of exoribonucleases. The 5’-to-3’ decay can only occur after the removal of the 5’ m^7^G cap, which is catalyzed by the decapping enzyme Decapping 2 (DCP2).

In addition to the classical deadenylation-mediated mRNA decay pathway, recent advances highlight the importance of cotranslational mRNA decay, referred to as translation-dependent mRNA decay (**Figure 1**) (Zhang and Guo, 2017; Tuck et al., 2020; Biasini et al., 2021; Mishima et al., 2022). For a long time, ribosome disassociation was believed to be a prerequisite for mRNA degradation. However, in 2009 Hu and co-workers revealed that mRNA decay could occur on transcripts associated with translating ribosomes (Hu et al., 2009). Subsequent high-throughput experiments revealed that cotranslational mRNA decay is widespread (Pelechano et al., 2015; Yu et al., 2016; Ibrahim et al., 2018). Cotranslational mRNA decay is carried out by components of the translation-dependent cytosolic mRNA surveillance pathways, such as nonsense-mediated decay (NMD), no-go decay (NGD), and no-stop decay (NSD), which are generally thought to remove aberrant transcripts with premature stop codons, transcripts with stalled ribosomes, and transcripts lacking a stop codon, respectively. Nevertheless, a growing body of evidence suggests an important role for these pathways in downregulating functional mRNAs in yeast, mammals, and plants (Łabno et al., 2016; Yu et al., 2016; Simsek et al., 2017; Zhang and Guo, 2017; Heck and Wilusz, 2018; Tuck et al., 2020; Biasini et al., 2021; Morris et al., 2021; Mishima et al., 2022). It is becoming clearer that these translation-dependent mRNA surveillance pathways do not uniquely function in mRNA quality control, but also play an important role in downregulating a large spectrum of functional mRNAs at the transcriptome level. It remains to be determined how translation-independent and translation-dependent mRNA decay are coordinated during development and under stress conditions. In future, it will be interesting to test how classical and cotranslational mRNA decay processes contribute to the degradation of photosynthetic transcripts during ETI.

## Photosynthetic mRNA translation during ETI

During PTI, translation is globally reprogrammed, resulting in poor correlation with changes in mRNA levels (Xu et al., 2017). To date, changes of the translatome during ETI have been less studied than those of the transcriptome. With TRAP-seq (translating ribosome affinity purification RNA-seq), one group measured ETI-induced translatome changes at 2 h post AvrRpm1 induction, which triggers RPM1-dependent ETI (Meteignier et al., 2017). No global translational reprogramming was observed during ETI. At the given time point, only three photosynthesis-related differentially expressed genes (DEGs) were found to be downregulated, namely *PSAD-2*, *LHCB2.2* and *LHCB4.2*. However, differentially translated genes (DTGs) for photosynthesis were highly represented among the 2591 down-regulated DTGs (Meteignier et al., 2017). Hardly any photosynthetic genes were found among the upregulated DEGs and DTGs. Based on this 2 h ETI (early stage) dataset the high number of DTGs and low number of DEGs among photosynthetic transcripts suggest that most of the photosynthetic transcripts are disassociated from polysomes and transferred into RNA-processing bodies for decapping or for short-term storage. Thus, it is highly possible that most photosynthetic transcripts undergoing mRNA decay eventually do so in a classical manner, a promising hypothesis worth testing in the future.

Using Ribo-seq, a protocol entailing polysome enrichment, RNase I digestion, and cDNA library construction to recover ribosome-protected mRNA fragments for further analysis, the work by Yoo et al. measured the ETI-induced traslatome at 8 h post inoculation with *Pseudomonas syringae* pv. maculicola expressing AvrRpt2, which triggers RPS2-dependent ETI. In this study, 983 upregulated DEGs, 203 downregulated DEGs, 926 upregulated DTGs and 156 downregulated DTGs were discovered (Yoo et al., 2020). At the time point after treatment in this study (8 h post inoculation, approximately middle stage of ETI), ETI-induced changes in transcription and translation are highly correlated (r = 0.92). Consistent with results at 2 h of AvrRpm1-triggered ETI (Meteignier et al., 2017), DEGs and DTGs for both *LHCB2.2* and *LHCB2.4* were also downregulated (Yoo et al., 2020). Interestingly, photosynthetic transcripts showed varied translation efficiency, with some displaying increased and some decreased efficiency at 8 h after AvrRps2-triggered ETI, though both their DEGs and DTGs were downregulated. For example, the reduction for *LHCB2.2* was 2.8-fold by RNA-seq, while it was 2.1-fold by Ribo-seq. This means that polysome-bound *LHCB2.2* mRNA decreased less compared to the total decrease of *LHCB2.2* mRNA, i.e. polysome-bound *LHCB2.2* transcript contributed less to mRNA decay than unbound transcripts or those bound to free 40S or 80S ribosomes. Conversely, the reduction for *LHCA6* was 1.5-fold by RNA-seq, while it was 1.9-fold based on Ribo-seq, suggesting that polysome-bound *LHCA6* mRNA contributed more to mRNA decay, indicating that translation-dependent decay of *LHCA6* mRNA occurs. The varying translation efficiency suggests that translation-dependent and -independent mRNA decay contribute differently to the regulation of different photosynthetic transcripts.

Two major caveats in these considerations are that we are comparing translatome datasets from two different groups, which were generated with different materials, and even with different translatome analysis methods, and that these datasets were derived from single, and different, time points after ETI induction with two different effectors. For a comprehensive understanding of ETI-induced translatome changes, more time points across the whole ETI process are required.

## Formation of Rubisco-containing bodies during ETI

Rubisco (Ribulose-1,5-biphosphate carboxylase-oxygenase), the most abundant protein on earth, accounts for 12%-30% of total leaf protein in C3 plants (Andersson and Backlund, 2008). Rubisco-containing bodies (RCB), small spherical bodies localized both in the cytoplasm and vacuole, were first observed in naturally senescing wheat leaves by immunolocalization of the large subunit of Rubisco (Chiba et al., 2003). Later, RCBs were confirmed to be a type of autophagic body that transfers Rubisco and other stromal proteins to the vacuole for degradation (Chiba et al., 2003; Ishida et al., 2008; Wada et al., 2009). The formation of RCBs is always associated with carbon starvation or senescence, so it is considered to be an efficient nutrient recycling mechanism while maintaining some basal functions of chloroplasts (Izumi et al., 2010; Yoshitake et al., 2021).

RCB induction was also observed during AvrRps4-triggered ETI (Dong and Chen, 2013). The avirulent strain *P. syringae* pv. *tomato* DC3000 expressing AvrRps4 [DC3000(AvrRps4)] induced many small RCBs but few large bodies. Conversely, the virulent strain DC3000 induced a small number of RCBs but proportionally more large bodies. The large bodies showed a similar size with chloroplasts, indicating these are possibly whole-chloroplast autophagic bodies. It seems that ETI preferentially induces RCB formation rather than whole-chloroplast autophagy, indicating that RCB formation might play a role during ETI. The authors found that RCB formation and ETI were abolished in *atg5-1*, a mutant defective in autophagy. Surprisingly, DC3000(AvrRps4)*-*induced cROS accumulation was also abolished in *atg5-1*, suggesting that RCB formation facilitates cROS accumulation. If this assumption is true, one would expect less cROS accumulation in response to virulent DC3000.

Rubisco catalyzes both carboxylation and oxygenation, two competing reactions involving CO_2_ and O_2_ as substrates, respectively (Andersson and Backlund, 2008). Carboxylation consumes both NAPDH and ATP, which are produced by the photosynthetic light reaction. Because NADP^+^ is reduced by an electron derived from the photosynthetic electron transport chain (PETC), Rubisco-mediated CO_2_ reduction relieves PSII excitation pressure under normal conditions, thus minimizing O_2_^•-^ production at PSI and Cytochrome b_6_f complex (Cyt b_6_f). On the other hand, Rubisco-mediated oxygenation consumes O_2_, and low O_2_ levels could also minimize cROS production. It is therefore reasonable to hypothesize that RCB-mediated mobilization of Rubisco out of chloroplasts not only reduces the PETC electron sink but also reduces O_2_ consumption, two processes that synergistically promote cROS accumulation. This could partially explain why ETI induces RCBs, but not whole-chloroplast autophagy. It is worth noting that other stromal proteins, such as glutamine synthetase (Chiba et al., 2003), a key enzyme in nitrogen assimilation, were also detected in RCBs. Blocking chloroplast nitrogen assimilation could also reduce NADPH consumption (Baslam et al., 2021), and thus enhance cROS accumulation.

## Discussion

Under normal conditions, chloroplasts are the center for carbon fixation and nitrogen assimilation. However, during ETI chloroplasts must be reprogrammed to be a center for production of ROS, defense-related hormones, and metabolites (Sowden et al., 2018; Kuźniak and Kopczewski, 2020; Kachroo et al., 2021; Littlejohn et al., 2021). We hypothesize that mRNA decay of photosynthetic transcripts, either translation-dependent or translation-independent, impairs the repair cycle of PSII and thus facilitates the generation of ROS such as ^1^O_2_, OH^•^ and H_2_O_2_ at PSII. It is possible that decay of nuclear-encoded transcripts alone will lead to an uncoupling of expression of nuclear-encoded and chloroplast-encoded proteins. However, we think this is unlikely since all six sigma factors (SIG1-SIG6) for expression of chloroplast-encoded genes are nuclear-encoded and their transcripts are also destabilized during ETI (Su et al., 2018; Hwang et al., 2022). Meanwhile, removing Rubisco and glutamine synthetase from chloroplasts by RCBs reduces the consumption of both NADPH and O_2_, causing stromal overreduction. Correspondingly, a highly reduced stroma would further exacerbate PSII excitation pressure and enhance electron leakage at the donor side of PSI or Cyt b_6_f to O_2_, resulting in O_2_^•-^ production. Together, these multi-layered reprogramming mechanisms lead to induction of high cROS accumulation during ETI. In addition to these events discussed above, other cytoplasmic events connected with HR development may also contribute to cROS accumulation. For example, it would be of interest to test whether ETI-induced cytoplasmic Ca^2+^ influx directly or indirectly affects chloroplast dynamics and thereby promotes cROS accumulation. In the future, it will be interesting to determine the impact of ETI on PSII functioning.

## Author contributions

JS wrote the draft. WG revised and edited the manuscript. All authors contributed to the article and approved the submitted version.
